# Multipath joint ablation strategy for focal atrial tachycardia originating from patent foramen ovale: a case report

**DOI:** 10.3389/fcvm.2024.1424187

**Published:** 2025-01-08

**Authors:** Fuqiang Liu, Yifei Li, Song Yan, Lijun Liu, Kaiyu Zhou, Yimin Hua

**Affiliations:** ^1^ Key Laboratory of Birth Defects and Related Diseases of Women and Children of MOE, Department of Pediatrics, West China Second University Hospital, Sichuan University, Chengdu, Sichuan, China; ^2^ Department of Nursing, West China Second University Hospital, Sichuan University, Chengdu, China

**Keywords:** atrial tachycardia, catheter ablation, tachycardia associated cardiomyopathy, patent foramen ovale, managing strategy, case report

## Abstract

**Introduction:**

Focal atrial tachycardia (FAT) is predominant in the pediatric population. Recent research has identified cases of sustained FAT originating from the interatrial septum (IAS); a subset of cases presents a unique challenge, with foci originating from the peri-patent foramen ovale (peri-PFO), requiring specialized management during catheter ablation. Here, we present a rare case of peri-PFO-associated FAT that resulted in tachycardia-related cardiomyopathy and propose a comprehensive multipath joint strategy for the successful treatment of PFO-associated FAT.

**Case presentation:**

A 10-year-old boy presented with refractory cardiomyopathy associated with incessant atrial tachycardia, unresponsive to metoprolol. A 12-lead electrocardiogram revealed a narrow QRS complex tachycardia with a rate of 157 beats per minute and a prolonged RP relationship. Echocardiography indicated a severely reduced ejection fraction of 22%. Subsequent electrophysiological study findings identified the tachycardia as originating from the anterior limbus of the PFO. Radiofrequency ablation was performed at the earliest activation site and surrounding structures, encompassing the right atrial septum, non-coronary sinus of Valsalva, and the left atrium peri-PFO. Post-procedure, the patient remained free from arrhythmia and showed restored normal cardiac function and was prescribed a low-dose β-blocker for 1 month. Remarkably, the patient continued to be well without the need for any medications for the subsequent 3 months.

**Conclusion:**

The structure of the PFO brought significant challenges in performing successful ablation. The multipath strategy was beneficial in managing peri-PFO-associated FAT based on its anatomical vicinity of the target.

## Introduction

Supraventricular tachycardia (SVT) represents a diverse group of arrhythmias originating at or above the His bundle and is commonly encountered in pediatric cases (1). In adults, atrial fibrillation is often the most prevalent form of SVT, while in children, atrioventricular nodal reentrant tachycardia (AVNRT), atrioventricular reentrant tachycardia (AVRT), and focal atrial tachycardia (FAT) predominate (2). Prolonged episodes of SVT, particularly FAT, can lead to tachycardia-induced cardiomyopathy, compromising cardiac function over time. While pharmacological therapy is typically employed to terminate or convert these arrhythmias, FAT can sometimes be resistant to drug-based treatments. For cases of symptomatic FAT in pediatric patients, catheter ablation has become an increasingly viable option. This procedure targets specific arrhythmogenic foci, which may be located in various anatomical sites such as the atrioventricular ring, crista terminalis, coronary sinus (CS) ostium, or a pulmonary vein (PV), offering a more definitive treatment (2, 3).

Recent studies have reported cases of sustained focal atrial tachycardia (FAT) arising from the interatrial septum (IAS), a rare phenomenon due to the IAS's predominant composition of connective tissue with minimal myocardial involvement (4). Ablating arrhythmogenic foci within the IAS presents notable challenges, especially when the focus is unilateral, which can lead to radiofrequency ablation failure (5). Consequently, bilateral ablation may be considered for FAT originating from the IAS. A particularly challenging subset of cases involves arrhythmic foci in the peri-patent foramen ovale (peri-PFO) region, which requires specialized management during catheter ablation procedures (6, 7). The dynamic motion of the valve of foramen ovale and the anatomical complexity of the peri-PFO tissue may compromise catheter stability, potentially reducing ablation success rates.

In this report, we describe a rare case of peri-PFO-associated FAT that progressed to tachycardia-induced cardiomyopathy. We detail the electrophysiological identification of peri-PFO FAT and propose a comprehensive, multi-path strategy for the effective termination of this arrhythmia.

## Case presentation

### Ethic approval

The study was approved by the ethics committee of the West China Second Hospital of Sichuan University (approval number: 2021-069). We also obtained written informed consent from the patient's parents prior to performing whole exon sequencing (WES) and for the inclusion of the patient's clinical and imaging details in this publication.

### Clinical presentation

We present the case of a 10-year-old male patient [weight 39 kg, body mass index (BMI) 18 kg/m^2^] who was admitted to our cardiac intensive care unit following a two-year history of progressive exertional dyspnea. He reported reduced tolerance for physical activity, palpitations, and pallor. His parents denied any additional symptoms such as fainting, cyanosis, fever, seizures, edema, vomiting, or abdominal discomfort. There was no history of chest pain, syncope, or recent viral infection. The patient had been suspected of having dilated cardiomyopathy and incessant sinus tachycardia a year prior and was treated with metoprolol, an angiotensin receptor neprilysin inhibitor, furosemide, and spironolactone.

Physical examination revealed a chronically ill appearance with marked diaphoresis and fatigue. Tachycardia was noted, with a heart rate of 150–160 beats per minute, along with irregular premature beats. Blood pressure was normal across all four extremities. Respiratory auscultation revealed coarse breath sounds and diffuse wheezing in both lungs, while the cardiac apex was displaced to the left lower quadrant, suggesting an enlarged heart. The heart rhythm was irregular with occasional premature beats, but no murmurs were detected. The abdomen was soft with no splenomegaly, and muscle strength and tension were normal in all limbs. No pathological signs were present, nor were there any signs of meningeal irritation.

The patient's past medical history was otherwise unremarkable, with no family history of cardiovascular disease, hypertension, or coronary artery disease. The absence of significant risk factors for cardiovascular conditions further complicated the clinical picture, prompting a detailed workup to determine the underlying cause of his symptoms.

### Laboratory and imaging examinations

The initial laboratory evaluation revealed normal results for routine blood cell counts, blood gas analysis, and hepatic and renal function tests. However, B-type natriuretic peptide (BNP) levels were significantly elevated at 4,356.8 pg/ml (normal range: <60 pg/ml), suggesting significant cardiac stress, although cardiac troponin I (cTnI) remained within normal limits. Screening for rheumatic disease, autoimmune conditions, and thyroid dysfunction was negative, and viral testing for pathogens such as SARS-CoV-2, Coxsackievirus, adenovirus, influenza, rhinovirus, and respiratory syncytial virus yielded negative results.

**Figure 1:**
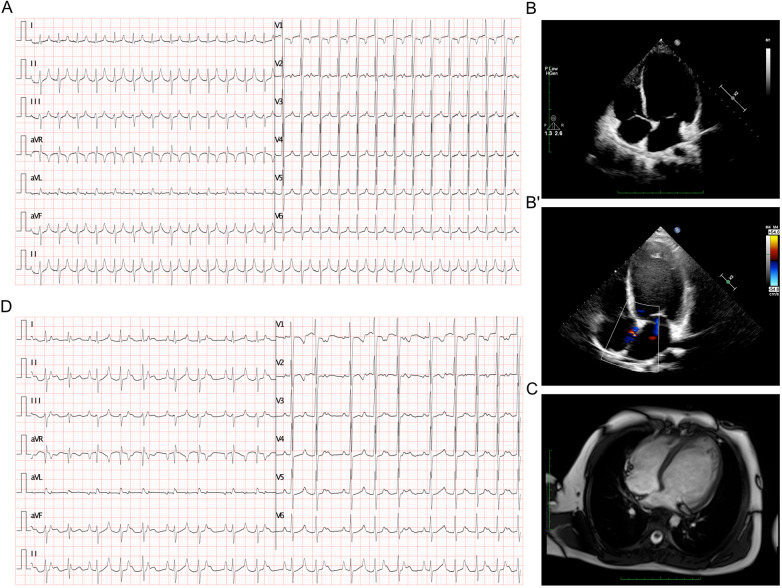
ECG and imaging presentation of the patient. **(A)** ECG presented a tachycardia at rate of 157 bpm. **(B-B’)** Echocardiography presented enlarged left atrium of 35 mm and left ventricle of 57 mm **(B)**, and a PFO **(B’)**. **(C)** Cardiac MRI presented a dilated left ventricle which was absent from any signaling myocardial fibrosis against the diagnosis of dilated cardiomyopathy. **(D)** ECG demonstrated an atrial rate of 124 bpm after amiodarone treatment, and Wenckebach block was identified which lead to a slower ventricular rate at about 82 bpm.

The 12-lead electrocardiogram (ECG) revealed a narrow QRS complex tachycardia at 157 beats per minute (bpm) without discernible *P* waves, but notable high-peaked T waves (Figure 1A). Echocardiography indicated severe systolic dysfunction with a left ventricular ejection fraction of 22%, an enlarged left atrium measuring 35 mm, and a dilated left ventricle measuring 57 mm (Figure 1B), with a patent foramen ovale (PFO) confirmed (Figure 1B'). The right atrium and ventricle appeared normal. Cardiac MRI supported the findings of left ventricular dilation but did not show evidence of myocardial fibrosis, ruling out the diagnosis of dilated cardiomyopathy (Figure 1C). After initiation of amiodarone therapy, ECG showed a reduction in the atrial rate to 124 bpm with the development of a Wenckebach block, resulting in a slower ventricular rate of approximately 82 bpm (Figure 1D). The biphasic *P* wave in lead V1 (−/+), along with positive, pointed *P* waves in leads I, II, III, and aVF and negative *P* waves in lead aVR (Figure 1D), pointed to a focal origin in the high right atrium, such as the superior vena cava or right atrial appendage. This pattern raised suspicion for inappropriate sinus tachycardia, though the atrial tachycardia persisted during hospitalization.

To further exclude a diagnosis of dilated cardiomyopathy, whole exome sequencing (WES) was performed to detect potential genetic variants. No cardiovascular-related mutations, particularly those affecting ion channels, were identified in the proband or his parents. Due to the severity of clinical symptoms and the failure of pharmacological conversion, the patient underwent an electrophysiological study with subsequent catheter ablation.

### Electrophysiologic study and ablation

**Figure 2:**
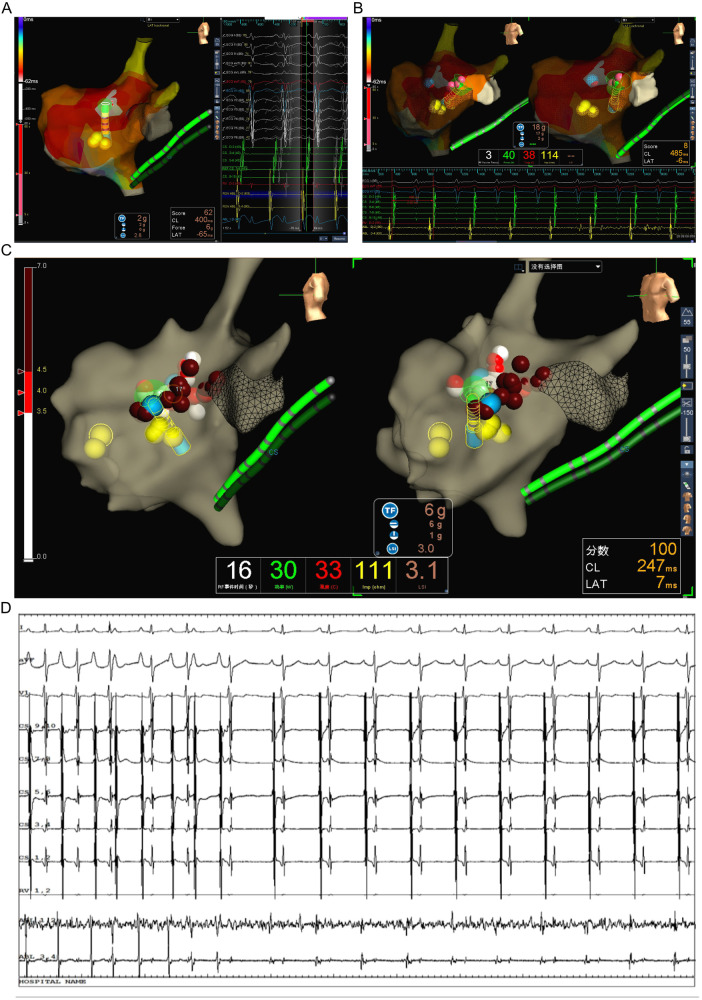
Identifying the originating of FAT in the patient. **(A)** Activating mapping suggested the earliest site at the interatrial septum in right atrium on left lateral view with EnSite Velocity system. **(B)** The catheter slipped into the left atrium through PFO and ablation performed at the anterior wall of left atrium. **(C)** The catheter retreated into the right side to continue the procedure at the vicinity of the earliest site along the limbus of PFO. **(D)** The tachycardia had been terminated and converted to sinus rhythm when ablation performing at the limbus of PFO.

**Figure 3:**
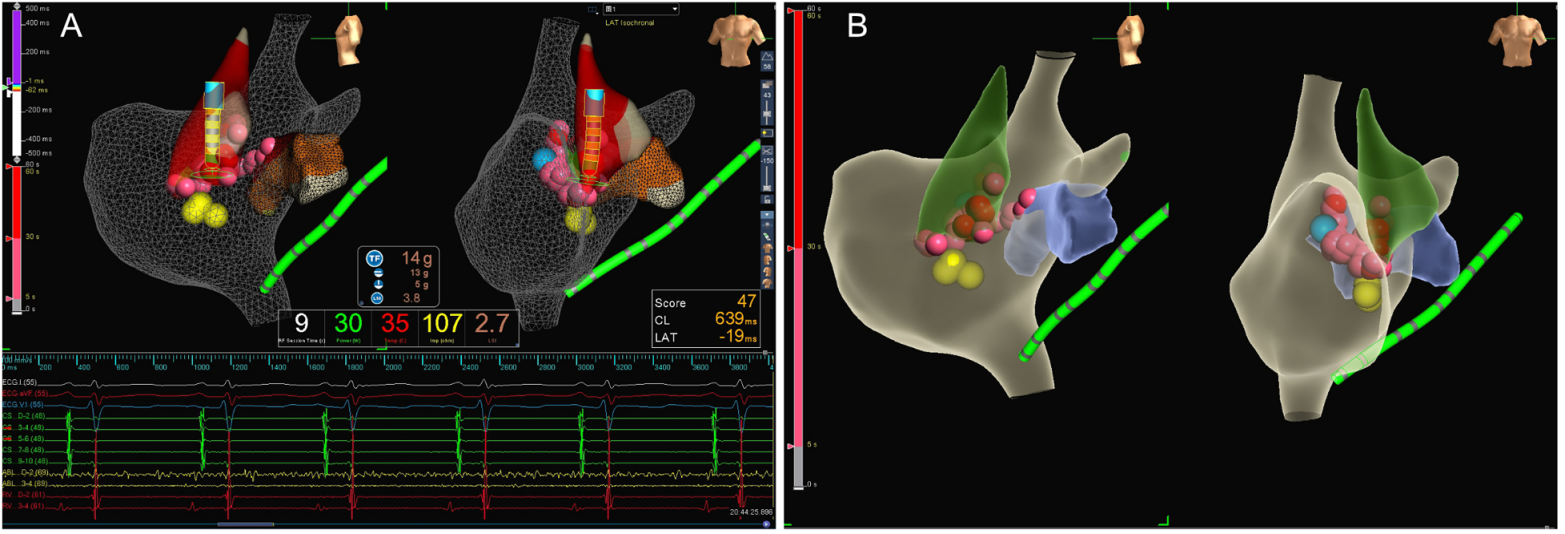
Multipath ablation strategy in the patient. **(A)** Ablation performed additional lesions in the non-coronary aortic sinus of Valsalva near to the earliest activate site in the right interatrial septum. **(B)** All ablation lesions for this patient, as the gray region is part of right atrium, the blue region is part of left atrium, and the green is part of noncoronary cusp.

**Figure 4:**
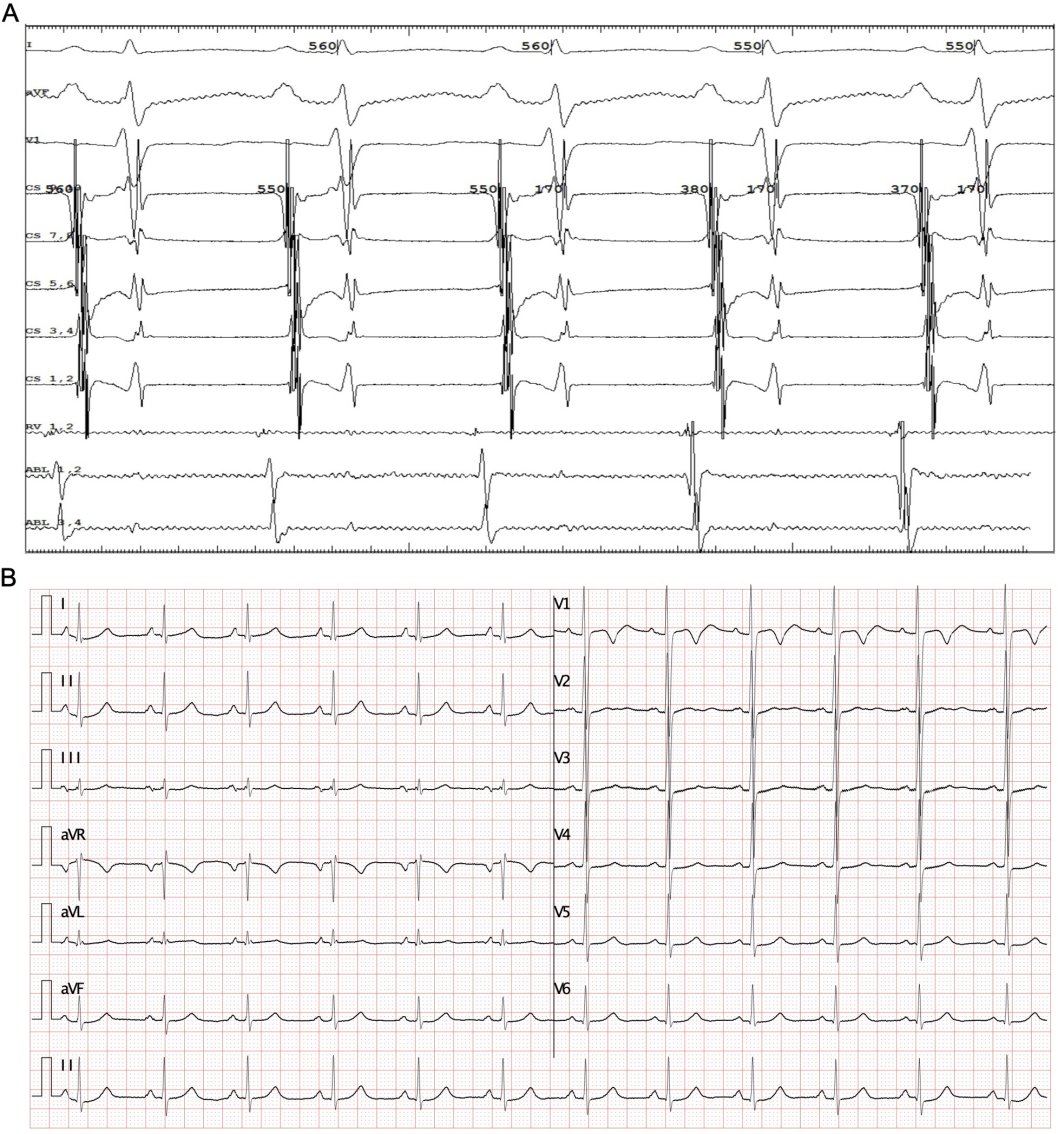
Electrocardiogram after ablation. **(A)** An Intracardiac electrocardiogram for the sinus rhythm, the ablation catheter placed at the target, and the right ventricular catheter was placed at high right atrium. **(B)** ECG with rate of 96 bpm after ablation, and AV 1:1 conducted.

The patient underwent the entire procedure under general anesthesia by inhaling 2%–3% sevoflurane continuously after initial anesthetic induction with propofol (2 ug/kg) and fentanyl (2 mg/kg). Diagnostic catheters [Inquiry^TM^ Steerable Diagnostic Catheter (Abbott Corp., St. Paul, MN, USA) for the coronary sinus, Supreme Electrophysiology Catheter for the His potential and Inquiry^TM^ Electrophysiology Catheter (Abbott Corp., St. Paul, MN, USA) for the right ventricle) were placed in the coronary sinus, the bundle of His, and the right ventricle, respectively, after successful puncture of both femoral veins and heparinization (100 U/kg). Initially, an incessant atrial tachycardia was underlined, and the earliest atrial signal appeared to be on the His catheter with an atrial cycle length of 350–420 ms with 1:1 conduction to the ventricle. Changes in the ventricular-atrial interval were observed which suggested the tachycardia was a kind of FAT. Fine mapping was performed with an irrigated ablation catheter (TactiCathTM Contact Force Ablation Catheter, Sensor EnabledTM, Abbott Corp., USA) through an SR0 sheath (Fast-CathTM Guiding Introducer SR0TM, Abbott Corp., USA) to identify the earliest atrial electrograms. The earliest atrial signals were 65 ms ahead of the surface *P* wave with QS unipolar electrograms and appeared to be an anterior limbus of PFO (Figure 2A). Radiofrequency energy was used at 30–40 W and 30 cc/min flow for 30–60 s per lesion at the earliest site with lesion size index (LSI) values of 3.5–4.0. During the ablation procedure, the tachycardia cycle length (TCL) extended to 460 ms. However, due to the anatomical characteristics of the mobile limbus of the PFO, optimal catheter-tissue contact was challenging to maintain, with contact force readings remaining suboptimal at 1–2 g (Figure 2A). Attempts to increase contact force resulted in inadvertent catheter displacement through the PFO into the left atrium. Next, we performed the procedure with the contact force for 10–20 g at 30 W and 30 cc/min flow for 30 s per lesion at the anterior wall of the left atrium opposite to the earliest site on the right side with LSI values of 4.0–4.5 (Figure 2B). The TCL was further extended to 480–500 ms during ablation on the left side. After that, we retreated the catheter into the right side to continue the procedure at the vicinity of the earliest site along the limbus of the PFO. Tachycardia was eventually terminated and converted to sinus rhythm (Figures 2C,D). Furthermore, additional lesions around this region that had been identified as the foci of FAT, including the opposite side of the earliest site in the non-coronary aortic sinus of Valsalva (Figures 3A,B) also received radiofrequency at a force of 10–20 g at 30 W and 30 cc/min flow for 30 s per lesion with LSI values of 4.0–4.5 to ensure enhanced treatment efficacy via multipath strategy. Then, standard atrial and ventricular stimulation protocols with single and double extra stimuli (S1S2 400/300 ms and S1S2S3 400/300/300 ms) and burst pacing (S1S1 200–300 ms) were used, and isoproterenol was repeatedly administered. The ablation electrode was ultimately positioned at the anterior limbus of the PFO, while the right ventricular electrode, serving as a high right atrial reference, demonstrated that the electrical signals at the high right atrium preceded those at the ablation target site (Figures 4A,B). Multiple stimulation protocols failed to induce any additional arrhythmias. The total procedure duration was 132 min, with a cumulative radiation exposure of 20 mGy. Activation mapping comprised 300 sampling points, predominantly concentrated in the RA. A total of 26 ablation lesions were delivered: 13 lesions surrounding the earliest activation site in the RA, 8 lesions in the left atrium (LA), and 5 lesions in the NCC following tachycardia termination.

### Final diagnosis and follow-up

The patient was diagnosed with focal atrial tachycardia FAT originating from the peri-PFO region, resulting in tachycardia-induced cardiomyopathy. Following successful catheter ablation, the patient was initiated on a medical regimen comprising clopidogrel (0.5 mg/kg/day once daily) for antiplatelet therapy, along with heart failure management including metoprolol (1 mg/kg/day divided twice daily), sacubitril/valsartan (2 mg/kg/day divided twice daily), and spironolactone (1 mg/kg/day divided twice daily) to address left ventricular dysfunction and chamber dilation. At his 6-month follow-up, the patient reported resolution of his prior symptoms. A 12-lead electrocardiogram confirmed normal sinus rhythm at 80 beats per minute. Echocardiography demonstrated an improvement in left ventricular function, with the ejection fraction normalized to 60%, and the left ventricular end-diastolic diameter reduced to 46 mm. The patient's medication regimen was subsequently discontinued. At 6 months post-ablation, repeat ECG and echocardiographic evaluations remained within normal limits. There was no complication and adverse event observed for the follow-up period.

## Discussion

FATs can arise from various locations within both atria, with up to 63% originating from structures in the right atrium, including the tricuspid annulus, crista terminalis, and CS ostium. Conversely, 37% have been identified to emerge from left atrial structures such as the pulmonary veins, mitral annulus, and CS body (8). In rare instances, FAT may have its origin in the atrial appendage, IAS, and/or the non-coronary cusp (9, 10). Various tachycardias may lead to TIC, marked by cardiac enlargement, decreased cardiac function, and the onset of heart failure. Pediatric patients, in particular, warrant screening for secondary factors. In this report, we present a rare pediatric case involving FAT originating from the limbus of a PFO. To our knowledge, this is the first detailed documentation of FAT originating from the limbus of PFO in a pediatric patient, demonstrating TIC, and subsequently recovering normal sinus rhythm and cardiac function following successful radiofrequency ablation. A similar case was reported by Tan et al. in an adult patient (7), and Siddiqui et al. briefly described eight cases of FAT originating from the limbus of a PFO, with seven cases having their origin on the left IAS and one on the right IAS of the PFO (11).

A PFO represents a persistent fetal interatrial communication resulting from incomplete fusion of the septum primum to the septum secundum, typically in the posteroinferior portion of the medial atrial wall. The prevalence of PFO is estimated to be approximately 25%–30% within the population (4). Detection of PFO can be challenging with transthoracic echocardiography (TTE) alone, as this method exhibits limited sensitivity. Specifically, TTE using color flow Doppler has a sensitivity of 21.4%, whereas the use of bubble contrast increases sensitivity to 53.8% for detecting PFO (12). In our patient, PFO was identified through TTE, and subsequent 3D electroanatomical mapping confirmed it to be the origin of atrial tachycardia. Embryologically, the central region of the foramen ovale typically consists of a membranous structure predominantly composed of thin fibrotic tissue devoid of action potential, with myocardial fibers and automaticity being either scattered or absent (13, 14). The presence of PFO is recognized as a significant risk factor for cryptogenic stroke, particularly in young adults where paradoxical embolism serves as a primary mechanism. This risk may be heightened during procedures involving peripheral venous access, including radiofrequency catheter ablation (RFCA) (15, 16). Based on current guidelines and the patient's asymptomatic status, we implemented a conservative management strategy with systematic follow-up, deferring PFO closure. Recent advancements in 3D electroanatomical mapping have confirmed these histological characteristics (17). Despite the rarity of arrhythmias originating from this central area owing to the lack of myocardial fibers, adjacent atrial muscle bundles may still exert an arrhythmogenic effect. Focal mapping using a multielectrode diagnostic catheter could enhance the accuracy of localizing the site of the arrhythmogenic focus during catheter mapping and likely reduce the duration of the whole procedure. Ablation around the unclosed foramen ovale poses challenges, particularly in placing the catheter to the limbus of the PFO, which may be free. This may be the reason for the extended TCL after our first attempt. Ablation performed in the anatomical vicinity of the target could potentially increase the success rate of the ablation procedure. We performed radiofrequency in our patient by using a combined strategy in applying ablation in the RA, LA, and NCC through arteriosus and venous routine for the values of LSI for ablation at the right septal lower than that at the other positions. As known, the LSI, similar to ablation index (AI), is a dimensionless contact force parameter that allows for an accurate estimation of lesion volume in real time by integrating contact force (grams), duration (seconds) and power (watts), and the value of LSI or AI could predict successful ablation of various arrhythmias (18–20).

FAT originating from the IAS is more commonly observed in adult patients, with approximately half of these cases associated with structural abnormalities, including atrial septal defects and PFO (4). Septal AT can originate from either side of the septum, presenting distinct ECG manifestations that require careful differentiation. The *P* wave polarity in lead V1 has been shown to accurately predict the side of origin within the IAS (4). Literature demonstrates that the prevalence of atrial arrhythmias, particularly AF and AFL, associated with atrial septal defects (ASDs) increases with age. In unoperated adults, the estimated incidence of atrial arrhythmias is approximately 10% in patients under 40 years of age, increasing to at least 20% in older patients with pulmonary arterial hypertension and systemic hypertension (5, 21, 22). Paradoxically, ASD closure has been associated with a significant increase in atrial arrhythmia incidence. Among patients older than 40 years, approximately 40%–60% develop atrial arrhythmias within 10 years post-ASD closure (5). While ASD closure is associated with a higher prevalence of arrhythmias, no significant changes in arrhythmia prevalence have been observed in PFO patients between pre- and post-closure periods. Notably, there is no conclusive evidence suggesting an increased risk of ventricular arrhythmias in patients with either ASDs or PFOs, and no sudden cardiac death has been reported in this population. Mechanistically, it is believed that left-to-right shunting promotes cardiac remodeling secondary to chronic hemodynamic overload, resulting in focal interstitial fibrosis that creates a substrate for unsustainable or minimal reentry circuits, ultimately leading to atrial arrhythmias (23, 24). Consequently, closure is recommended for patients with non-reversible atrial arrhythmias.

Management of new-onset post-procedural arrhythmias is crucial (25). In PFO patients, a subset experiences transient arrhythmia during the initial days to weeks following the procedure, primarily attributed to local tissue irritation (26). Notably, up to two-thirds of these patients experience resolution of arrhythmias within one-year post-procedure. Due to the limited number of PFO patients requiring surgical repair, data regarding associated complications remains sparse. In contrast, surgical treatment of ASDs has been shown to increase the incidence of atrial arrhythmias through atriotomy-related electrophysiological remodeling (27, 28). Importantly, persistent arrhythmias may be directly related to the closure device itself. A significant consideration is that the defect itself represents a pre-existing potential central barrier of non-conduction for reentrant circuit formation (29). Additionally, device-related inflammation and scarring warrant careful monitoring.

The use of deflectable introducers can enhance catheter-tissue contact at the PFO limbus or ASD, particularly in post-device closure cases (30, 31). However, given the complex anatomical considerations, multiple-site ablation may be necessary to achieve optimal therapeutic outcomes. Various approaches exist to address these challenges, with transesophageal echocardiography or intracardiac echocardiography (ICE) proving particularly valuable in device closure patients (32), especially for pre-procedure onset arrhythmias. In our experience, the jugular approach may present operational challenges and increased procedural risks, particularly in pediatric cases. Recent technological advances in AF ablation, including PFA and very high-power short-duration (vHPSD) radiofrequency ablation, may improve success rates for ATs arising from PFO (33). ICE can enhance target visualization while reducing radiation exposure. A minimally fluoroscopic approach (MFA) should be pursued to minimize potential long-term adverse effects of radiation exposure, particularly in pediatric populations (34).

Our report has some limitations. The new mapping after TCL changed for the first ablation attempt to distinguish whether there were any other potential sites for ATs. Furthermore, we failed to carry out electrophysiological studies before the NCC ablation, which might have increased the procedure risk and prolonged the duration of x-ray exposure. In addition, we had not use ICE because we had no smaller size ICE catheter such as 8F ICE, but the 10F catheter we had may injure the child's femoral vein seriously.

## Conclusion

Our report presents a rare case of TIC in a pediatric patient, driven by incessant FAT originating from the peri-PFO. Catheter ablation proved to be an effective treatment strategy, offering the potential for long-term resolution without the need for antiarrhythmic medication. However, the unique anatomical structure of the PFO presented significant technical challenges during the ablation procedure. Based on this experience, we propose a multipath approach to the management of peri-PFO-associated FAT, emphasizing the importance of targeting the anatomical vicinity for successful ablation.

## Data Availability

The datasets presented in this study can be found in online repositories. The names of the repository/repositories and accession number(s) can be found in the article/Supplementary Material.
